# Acute association between heatwaves and stillbirth in six US states

**DOI:** 10.1186/s12940-022-00870-y

**Published:** 2022-06-16

**Authors:** Megan Richards, Mengjiao Huang, Matthew J. Strickland, Andrew J. Newman, Joshua L. Warren, Rohan D’Souza, Howard H. Chang, Lyndsey A. Darrow

**Affiliations:** 1 School of Public Health, University of Nevada, 1664 N Virginia St, m/s 0275, NV 89557 Reno, USA; 2 National Center for Atmospheric Research, Boulder, CO USA; 3 Department of Biostatistics, Yale University, New Haven, CT USA; 4 Rollins School of Public Health, Emory University, Atlanta, GA USA

**Keywords:** Heatwaves, Ambient temperature, Stillbirth, Fetal death

## Abstract

**Background:**

Heatwaves are becoming more frequent and may acutely increase the risk of stillbirth, a rare and severe pregnancy outcome.

**Objectives:**

Examine the association between multiple heatwave metrics and stillbirth in six U.S. states.

**Methods:**

Data were collected from fetal death and birth records in California (1996–2017), Florida (1991–2017), Georgia (1994–2017), Kansas (1991–2017), New Jersey (1991–2015), and Oregon (1991–2017). Cases were matched to controls 1:4 based on maternal race/ethnicity, maternal education, and county, and exposure windows were aligned (gestational week prior to stillbirth). County-level temperature data were obtained from Daymet and linked to cases and controls by residential county and the exposure window. Five heatwave metrics (1 categorical, 3 dichotomous, 1 continuous) were created using different combinations of the duration and intensity of hot days (mean daily temperature exceeding the county-specific 97.5^th^ percentile) during the exposure window, as well as a continuous measure of mean temperature during the exposure window modeled using natural splines to allow for nonlinear associations. State-specific odds ratios (ORs) and 95% confidence intervals (CI) were estimated using conditional logistic regression models. State-specific results were pooled using a fixed-effects meta-analysis.

**Results:**

In our data set of 140,428 stillbirths (553,928 live birth controls), three of the five heatwave metrics examined were not associated with stillbirth. However, four consecutive hot days during the previous week was associated with a 3% increase in stillbirth risk (CI: 1.01, 1.06), and a 1 °C average increase over the threshold was associated with a 10% increase in stillbirth risk (CI: 1.04, 1.17). In continuous temperature analyses, there was a slight increased risk of stillbirth associated with extremely hot temperatures (≥ 35 °C).

**Discussion:**

Most heat wave definitions examined were not associated with acute changes in stillbirth risk; however, the most extreme heatwave durations and temperatures were associated with a modest increase in stillbirth risk.

**Supplementary Information:**

The online version contains supplementary material available at 10.1186/s12940-022-00870-y.

Stillbirth is a rare, severe birth outcome that affects 1 out of every 160 births in the United States [1]. Stillbirths are more common among women who have advanced maternal age, high blood pressure, diabetes, or who are obese, as well as among those who smoke during pregnancy [2–6], but often times no contributing cause is identified. There is a growing effort to identify risk factors for stillbirth, such as environmental exposures including air pollution and ambient air temperature [7]. Since the 1980s the average global temperature has been steadily increasing, which has led to an increase in the number of heatwaves experienced each year [8]. Heatwaves have been associated with a variety of adverse health effects, including preterm and stillbirth, but comparisons across studies are limited because there is no gold standard definition of a heatwave.

A systematic review of 12 studies reported that exposure to hot temperatures during pregnancy may increase the risk of stillbirth; however, due to heterogeneity of methods, the authors were unable to pool results [9]. Studies differed in their exposure windows, parameterization of temperature, statistical methods, and definition of stillbirth. The three most recent studies done in North America all used a case-crossover approach. In Quebec (1981–2011, *n* = 5047), Auger et al. reported that the odds ratio for a temperature of 28 °C the day before death, compared to 20 °C, was 1.16 (CI: 1.02, 1.33) [10]. Basu et al. reported that in California (1999–2009, *n*= 8510) for every 10°F increase in apparent temperature in the week preceding stillbirth there was a 10.4% (CI: 4.4–16.8%) increased risk of stillbirth [11]. Using data from 12 sites across the United States (2002–2008, *n* = 447), Ha et al. reported a 6% (CI: 3.0–9.0%) increase in the risk of stillbirth from May–September associated with every 1 °C increase during the week preceding delivery [12]. However, these three studies were relatively small which led to less precision in effect estimates and an inability to examine the more extreme and rare heat events. Additionally, there is a lack of research regarding the physiologic mechanism that increases the risk of stillbirth during extreme temperature events, but it has been hypothesized that extreme heat reduces placental blood flow and increases dehydration which could precipitate fetal death [13].

We estimated associations between acute ambient temperature and stillbirth using fetal death records from six states, pooling results using meta-analytic techniques. To control for the seasonality of conceptions, we used a case–control approach and aligned the gestational exposure window of interest between the cases and controls. We used three different heatwave definitions based on the number of hot days over the threshold during the exposure window to determine if conclusions were different based on the definition. Additionally, we assessed continuous temperature to examine if the risk of stillbirth increased as the mean temperature during the exposure window increased, regardless of whether the exposure window contained a heatwave.

## Methods

### Study population

Data were collected from all singleton fetal death and birth records in California (1996–2017), Florida (1991–2017), Georgia (1994–2017), Kansas (1991–2017), New Jersey (1991–2015), and Oregon (1991–2017). Years of data differed based on availability of fetal death records. Stillbirth was defined as a fetal death that occurred after 20 weeks gestation and was issued a fetal death certificate. Information collected from the fetal death and birth records included date of delivery, estimated gestational age, maternal age, education, race, ethnicity, and county of residence, Stillbirths and live births were excluded from the current study if gestational age and last menstrual period (LMP) date were missing or if the recorded gestational age was not between 20–44 weeks. To avoid the fixed cohort bias, which can occur when the study population is defined by birth date, we defined the study population based on LMP date and limited our sample to women whose LMP was between September 1^st^ of the year prior to data availability (20 weeks prior to first possible birth) and February 28^th^of the last year of data availability (44 weeks prior to last possible birth), and did not restrict analyses to the warm season [14]. For example, in California, where data were available from 1996–2017, we included all births with an LMP date between September 1, 1995 – February 28, 2017.

### Study design

We implemented a matched case–control design because of concerns about confounding by seasonal patterns of conception [15]; our internal simulations indicated that a case–control approach with matched gestational timing of the exposure windows would be less susceptible to this bias than a case-crossover approach. Stillbirths were matched 1:4 to live births on maternal race/ethnicity (white, non-Hispanic; black, non-Hispanic; Hispanic; other; missing), maternal education (less than high school; high school degree; some college; college degree or more; missing), and county. If four controls were not available, cases were matched to as many controls as possible. The exposure window for the stillbirths was the 6 days prior to delivery and the date of delivery (lag 0–6). The exposure window for the controls was the gestational week corresponding to the matched stillbirth’s exposure window, calculated by adding the case’s gestational age to the LMP of the matched control. Meteorology was assigned by county, a matching factor, such that estimated associations would be driven by temporal contrasts of exposure. In Oregon, maternal race information on the fetal death records was not included in the data transfer; therefore, cases and controls were not matched on race/ethnicity, and race/ethnicity was not adjusted for in the Oregon analysis.

### Meteorologic data

Meteorologic data were collected for 1991–2017 from Daymet [16]. Daymet is a well-tested gridded meteorology dataset that uses ground-based in situ station observations and a collection of interpolation and regression algorithms to produce 1 km x 1 km gridded estimates of daily temperature and moisture, among other variables [17–19]. County-level temperatures were calculated by using the unweighted average of all grid cell estimates within a county. County-level temperature information was then linked to fetal death and birth records based on the reported maternal county and exposure window.

### Exposure definitions

#### Heatwave definitions

Heatwaves were defined based on the mean daily temperature using the relative temperature threshold framework, where a hot day was defined as any day that the mean temperature was above a given threshold, which for this study was the county-specific 97.5^th^percentile. The thresholds were county-specific because of climate differences both within and across states, and were defined using our full data period (1991–2017). Three heatwave definitions were created using the number of hot days in the previous week and the temperature over the threshold during the exposure window (lag days 0–6) [20]. Heatwave definition 1 (HW1) was a measure of the total number of hot days in the previous week, categorized as 0, 1, 2, and ≥ 3. Heatwave definition 2 (HW2) aimed to measure the impact of sustained heat and was defined as the number of consecutive hot days in the previous week. Separate indicator variables were created for ≥ 2 consecutive, ≥ 3 consecutive, and ≥ 4 consecutive days (i.e., if an exposure window had ≥ 4 consecutive hot days, it would also have ≥ 2 and ≥ 3 consecutive hot days). HW2 was defined using indicator variables, as opposed to exclusive categories, to allow for comparisons to previous literature. The final operationalization, heatwave definition 3 (HW3), was a continuous measure that incorporates both duration and intensity of hot days in the previous week, similar to an area under the curve measure. HW3 is the average difference between daily temperatures and the threshold during the exposure window; if the average was below the threshold, HW3 was given a value of 0.

#### Temperature definitions

In addition to the three heatwave definitions based on the temperature above a county-specific threshold, the 7-day average temperature was used to create one absolute measure and one relative measure of continuous temperature. The first was a measure of the 7-day average temperature during the exposure window; a categorical variable was also created with cut points to ensure sufficient sample size in each category (< 5 °C, 5–10 °C, 10–15 °C, 15–25 °C (REF), 25–27 °C, ≥ 27 °C). Second, because of the potential for acclimatization to local temperature norms, we assigned county-level percentiles to the 7-day average temperature based on the temperature data from 1991–2017; these were also categorized (< 2.5%, 2.5–10%, 10–25%, 25–75%, 75–90%, 90–97.5%, ≥ 97.5%).

### Statistical analysis

State specific odds ratios (ORs), 95% confidence intervals (CI), and variance–covariance matrices were estimated using conditional logistic regression models adjusting for maternal age (10–19; 20–24; 25–29; 30–34; 34–39; ≥ 40 years), LMP month (to control for recurrent seasonal trends), and LMP year (to control for long term trends). HW1 was modeled as a categorical exposure (0 days (REF), 1, 2, ≥ 3 days). HW2 was estimated using three separate models with binary exposures (yes/no: ≥ 2 consecutive days; ≥ 3 consecutive days; ≥ 4 consecutive days). HW3 was modeled as a continuous exposure, estimating the OR corresponding to a 1 °C increase over the threshold in the previous week. Results were stratified by the timing of the stillbirth (early (< 28 weeks) v. late (≥ 28 weeks, including term stillbirths)) and maternal race/ethnicity (white, NH; black, NH; Hispanic; other) was assessed using stratified analyses. We chose to examine stillbirths based on gestational age at delivery because it is thought that early stillbirths are difficult to prevent without early intervention and are more commonly due to genetic abnormalities [2, 21]. Continuous temperature and percentile were modeled using natural cubic splines to allow for nonlinear patterns. Placement and number of knots were selected based on cut-off values for the categorical variables and the distribution of absolute temperatures across states. Knots for continuous temperature were placed at 5, 20, and 25 degrees; continuous percentile knots were placed at 2.5, 10, 25, 75, 90, and 97.5 percent. As a sensitivity analysis, categorical parameterizations of continuous temperature and percentile were used and results were compared to those from the spline models. State-specific estimates were combined using a multivariate fixed-effect meta-analysis to estimate the average association across states. Specifically, we calculated a weighted average of state-specific vectors of log odds where the weight corresponds to the inverse of their corresponding variance–covariance matrices. Statistical analyses were completed using SAS 9.4 and R.

## Results

There were 140,428 stillbirths in the study (California = 51,577; Florida = 36,228; Georgia = 27,720; Kansas = 4,719; New Jersey = 15,046; Oregon = 5,138); these stillbirths were matched to 553,928 live births (Table 1). The distribution of matched variables varied slightly between cases and controls based on the number of matched controls per case. For example, in Florida there was an average of 3.89 controls per case, whereas in California there was an average of 4.0 controls per case. In Florida and Georgia, cases were more likely to have four controls if they were not missing race or education information. The distribution of race/ethnicity among matched sets varied across states; California had a higher proportion of Hispanic matched sets, Georgia had a higher proportion of black, non-Hispanic matched sets, and all other states had the largest proportion of matched sets reporting a maternal race/ethnicity of white, non-Hispanic. Older maternal age, which was not a matching factor, was more common among stillbirths than live births. In California, Kansas, and Oregon, there was a higher proportion of reported stillbirths born after 28 weeks (late stillbirth); in Florida, Georgia, and New Jersey there was a higher proportion of reported stillbirths born before 28 weeks (early stillbirth). The rate of stillbirth across study years in each state can be seen in Supplemental Table 1.

**Table 1:** Maternal, stillbirth (case), and live birth (control) characteristics, by state

	California (1996–2017)		Florida (1991–2017)		Georgia (1994–2017)		Kansas (1991–2017)		New Jersey (1991–2015)		Oregon (1991–2017)	
	Stillbirths *n* = 51,577	Controls *n* = 206,291	Stillbirths *n* = 37,009	Controls *n* = 144,107	Stillbirths *n* = 27,720	Controls *n* = 106,550	Stillbirths *n* = 4719	Controls *n* = 18,843	Stillbirths *n* = 15,046	Controls *n* = 60,214	Stillbirths *n* = 5138	Controls *n* = 20,546
Maternal Race/ethnicity^a^												
White, NH	25.9%	25.9%	38.8%	39.5%	35.3%	35.3%	63.8%	63.9%	38.4%	38.4%		
Black, NH	11.6%	11.6%	32.7%	32.0%	52.6%	52.2%	12.7%	12.6%	32.1%	32.0%		
Hispanic	49.6%	49.6%	25.5%	25.6%	8.2%	8.5%	18.8%	18.8%	22.3%	22.3%		
Other	11.6%	11.6%	1.7%	1.7%	2.6%	2.7%	3.8%	3.8%	6.4%	6.4%		
Missing	1.3%	1.3%	1.4%	1.2%	1.4%	1.4%	0.9%	0.9%	0.9%	0.9%	100.0%	100.0%
Maternal Education^b^												
Less than HS	26.6%	26.6%	20.4%	21.0%	3.4%	3.5%	20.4%	20.5%	14.8%	14.8%	21.6%	21.6%
High school	29.0%	29.0%	33.1%	33.9%	13.2%	13.8%	31.7%	31.7%	34.1%	34.1%	31.1%	31.1%
Some college	22.0%	22.0%	22.1%	22.7%	27.7%	28.9%	24.5%	24.5%	40.3%	40.2%	20.7%	20.7%
College degree	16.9%	16.9%	14.5%	14.8%	20.3%	21.2%	21.1%	21.1%			16.7%	16.7%
Missing	5.6%	5.6%	9.8%	7.6%	35.3%	32.7%	2.4%	2.3%	10.8%	10.8%	9.8%	9.8%
Maternal Age												
10–19 years	10.0%	10.1%	12.4%	12.4%	13.8%	14.0%	12.4%	12.7%	8.9%	9.6%	11.2%	11.5%
20–24 years	20.9%	23.5%	23.3%	26.7%	25.8%	28.1%	27.7%	29.2%	17.5%	20.1%	24.3%	27.2%
25–29 years	23.9%	26.7%	23.8%	26.6%	24.5%	25.9%	26.2%	28.1%	23.9%	26.1%	24.5%	27.4%
30–34 years	23.0%	23.7%	20.8%	21.1%	20.5%	19.7%	18.5%	20.0%	26.7%	27.0%	21.7%	21.7%
35–39 years	16.1%	12.8%	13.3%	10.6%	12.0%	10.0%	11.7%	8.3%	17.6%	14.0%	13.9%	10.0%
≥ 40 years	6.2%	3.2%	4.3%	2.7%	3.5%	2.4%	3.5%	1.6%	5.3%	3.1%	4.5%	2.3%
GA of stillbirth												
Early (< 28 weeks)	45.9%		53.4%		62.2%		37.7%		58.4%		41.6%	
Late (≥ 28 weeks)	54.1%		46.6%		37.8%		62.3%		41.6%		58.4%	

^a^ Oregon fetal death records did not contain information on maternal race, therefore race data could not be used

^b^ New Jersey only had three maternal education categories, with some college combined with college degree

Abbreviations: *GA* gestational age, *HS* high school, *NH* non-Hispanic

The average daily mean temperature across states from 1991–2017 was: California, 14.5 °C ± 7.4 °C; Florida, 21.57 °C ± 6.1 °C; Georgia, 17.67 °C ± 7.9 °C; Kansas, 12.76 °C ± 10.7 °C; New Jersey, 11.95 °C ± 9.5 °C; Oregon, 9.52 °C ± 7.2 °C (Table 2). The distribution of daily temperature is shown in Supplemental Figure 1. Table 2 shows the average number of days per year (across counties) that meet each heatwave definition. Across states, it was more common for counties to experience three or more hot days in the previous week than only two (HW1). The average number of days per year that had one, two, or three or more hot days in the previous week ranged from 7.9–11.4 days, 6.2–8.9 days, and 8.9–10.9 days, respectively. As HW2 became more extreme (i.e., the number of consecutive hot days increased), the average number of days classified as heatwaves decreased. There was an average of 3.5–5.3 days per year that had at least four consecutive hot days in the previous week, compared to 15.2–18.6 days per year that had at least two consecutive hot days in the previous week. There were on average 3.4–5.8 days per year in each county, across states, where the average degrees over the threshold (HW3) was greater than 0; for those days, the average degrees over the threshold during the exposure window ranged from 0.51–0.88 °C.

**Table 2:** County-level mean temperature and number of heatwave days per year, by state 1991–2017

	California	Florida	Georgia	Kansas	New Jersey	Oregon
Mean temperature (°C)	14.45 (7.4)	21.57 (6.1)	17.67 (7.9)	12.76 (10.7)	11.95 (9.5)	9.52 (7.2)
97.5^th^ percentile (°C)	25.80 (3.3)	29.05 (0.2)	28.47 (1.0)	29.14 (0.7)	26.84 (0.8)	22.26 (1.7)
HW1						
1 day	11.08 (7.3)	9.94 (7.5)	7.91 (6.8)	8.99 (6.2)	11.41 (6.9)	10.8 (7.4)
2 days	8.19 (5.8)	6.12 (5.8)	6.56 (6.1)	6.2 (5.0)	7.38 (6.3)	7.83 (5.5)
3 + days	10.91 (8.0)	10.06 (12.7)	10.79 (12.3)	10.24 (10.6)	8.86 (8.2)	9.53 (7.8)
HW2						
≥ 2 days	18.57 (10.7)	15.4 (15.9)	16.44 (15.3)	15.20 (12.8)	15.04 (12.1)	16.93 (10.8)
≥ 3 days	10.26 (7.7)	8.55 (11.1)	9.23 (10.8)	8.35 (9.3)	7.45 (7.1)	8.73 (7.2)
≥ 4 days	5.28 (5.3)	4.64 (7.3)	4.85 (7.0)	4.47 (6.4)	3.48 (4.2)	4.29 (4.6)
HW3^a^						
Days over 0	4.70 (5.1)	5.07 (7.8)	5.82 (8.1)	4.31 (7.2)	3.43 (4.2)	4.11 (4.7)
Mean^b^ (°C)	0.88 (0.8)	0.36 (0.3)	0.51 (0.4)	0.81 (0.7)	0.77 (0.6)	0.88 (0.7)

Values represent Mean(SD) across counties, within each state

^a^ HW3 is the average temperature over the 97.5^th^ percentile during the exposure window, if the value is negative it is set to 0

^b^ Mean HW3 value for days where the HW3 > 0

### Heatwave definitions

**Fig. 1:**
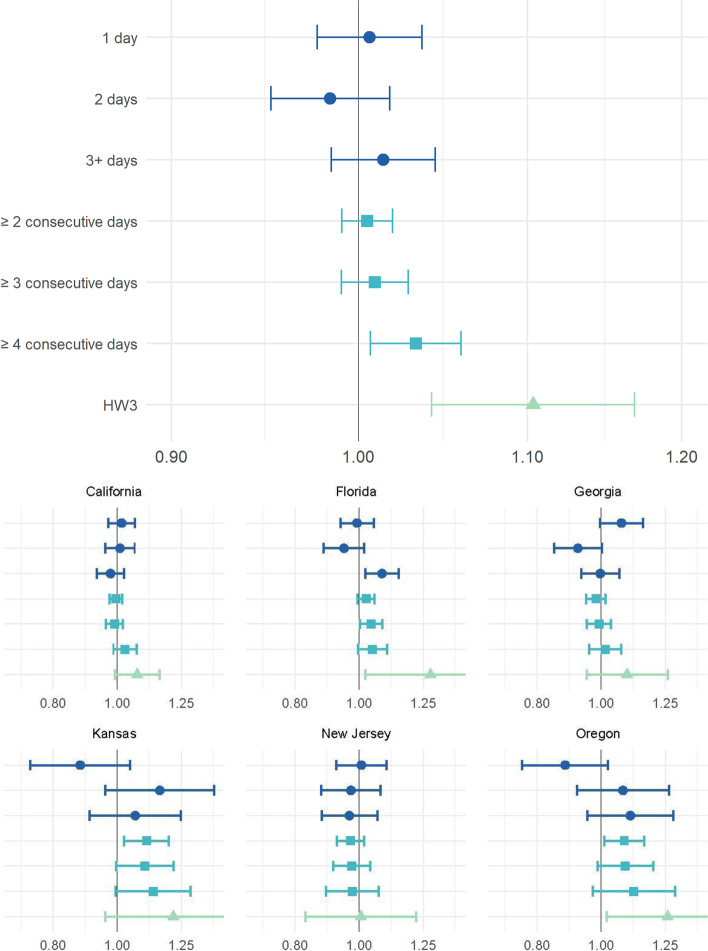
Odds ratios and 95% confidence intervals for the association between heatwaves and stillbirth in California, Florida, Georgia, Kansas, New Jersey, and Oregon. The reference category for heatwave definition 1 (dark blue lines, circles) is 0 hot days in the previous week. Heatwave definition 2 (blue lines, squares) are dichotomous exposure categories. Heatwave definition 3 (green line, triangle) presents the odds ratio associated with a 1° increase in the average degrees over the 97.5.^th^ percentile in the previous week. All models adjusted for maternal age, LMP month, and LMP year; cases matched 1:4 to controls based on maternal race/ethnicity, education, and county. Estimates shown in Supplemental Table 2

In pooled analyses, the estimated odds ratio increased as the heatwave definition became more extreme (Fig. 1, Supplemental Table 2). When a heatwave was defined using the total number of hot days in the previous week, there was not an increased risk of stillbirth (HW1). Similarly, defining a heatwave as at least two or three consecutive hot days in the previous week did not show an increased risk of stillbirth in pooled results. However, there was a slight increased risk of stillbirth associated with at least four consecutive hot days in the previous week [OR(CI): 1.03(1.00, 1.06)]. The odds ratio for the association between heatwaves and stillbirth was the strongest when the intensity and duration of the heatwave were taken into account (HW3). For every 1 °C increase in the 7-day average over the threshold, the odds of stillbirth increased by 10% (CI for OR: 1.04–1.17). State specific estimates were elevated in Florida, Kansas, and Oregon, but because of sample size were more imprecise than the other states (Supplemental Table 2). Estimates from New Jersey were compatible with a null effect for all heatwave definitions.

**Fig. 2:**
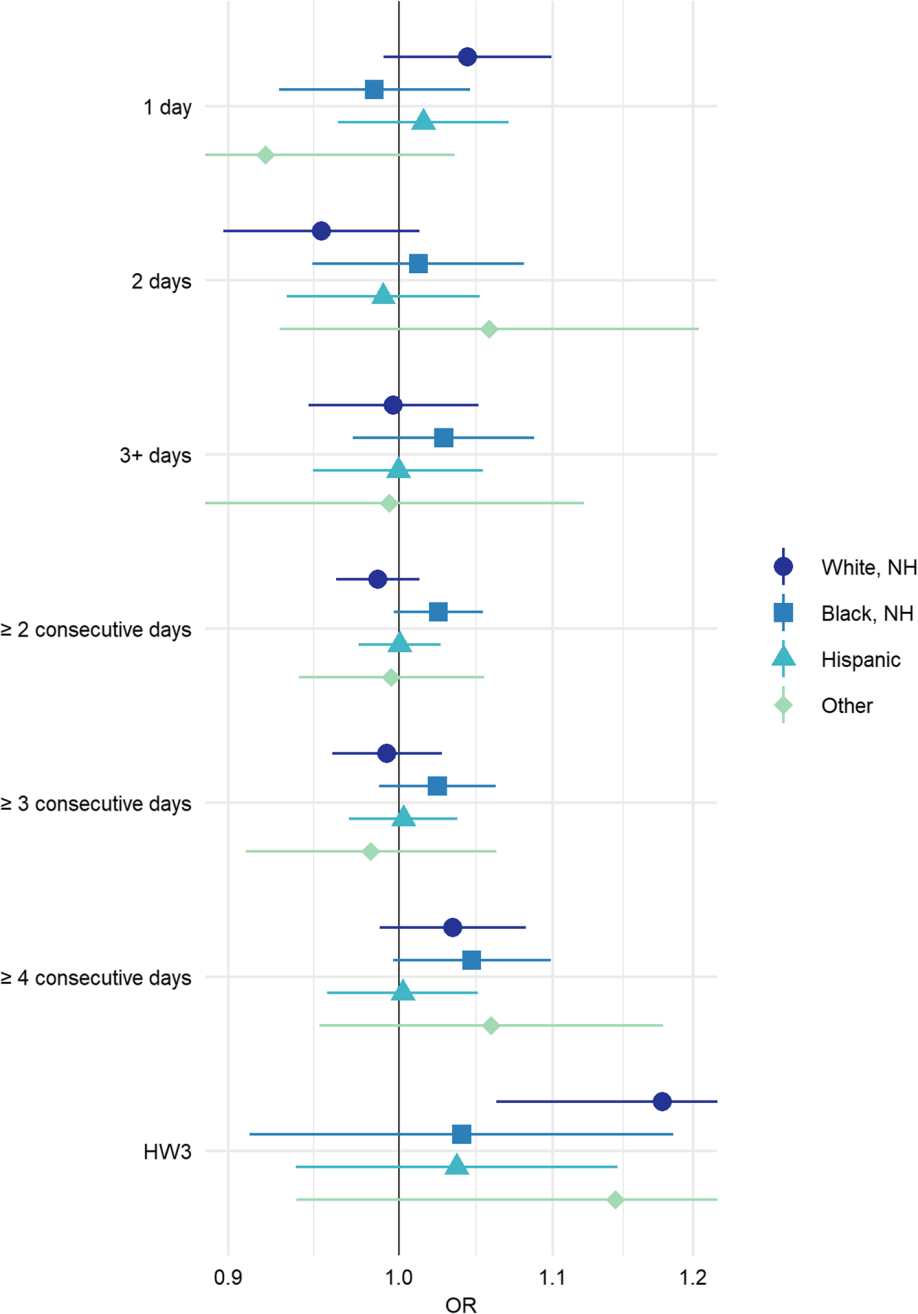
Odds ratios and 95% confidence intervals for the pooled association between heatwaves and stillbirth by maternal race/ethnicity. All models adjusted for maternal age, LMP month, and LMP year; cases matched 1:4 to controls based on maternal race/ethnicity, education, and county

**Fig. 3:**
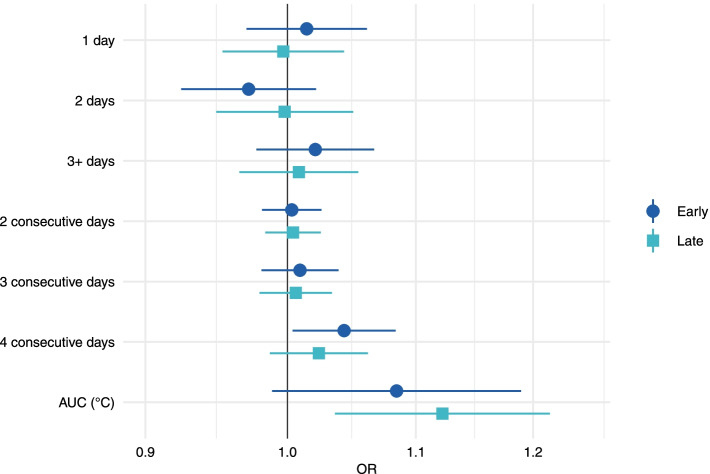
Odds ratios and 95% confidence intervals for the pooled association between heatwaves and stillbirth by the timing of the stillbirth. All models adjusted for maternal age, LMP month, and LMP year; cases matched 1:4 to controls based on maternal race/ethnicity, education, and county. Early stillbirth (< 28 weeks); Late stillbirth (≥ 28 weeks)

When pooled results were stratified, most heatwave definitions were compatible with a null effect. However, there was some evidence that the risk of stillbirth associated with the most extreme heatwave definitions was elevated for black, non-Hispanic women, although estimates were imprecise. For HW3, white, non-Hispanic women and women who were in the other race category had elevated ORs [OR(CI): 1.18(1.06, 1.30); OR(CI): 1.14 (0.93, 1.42), respectively], (Fig. 2, Supplemental Table 3). There was no evidence that the timing of the stillbirth (early versus late) affected results (Fig. 3, Supplemental Table 4.

### Continuous measures

**Fig. 4:**
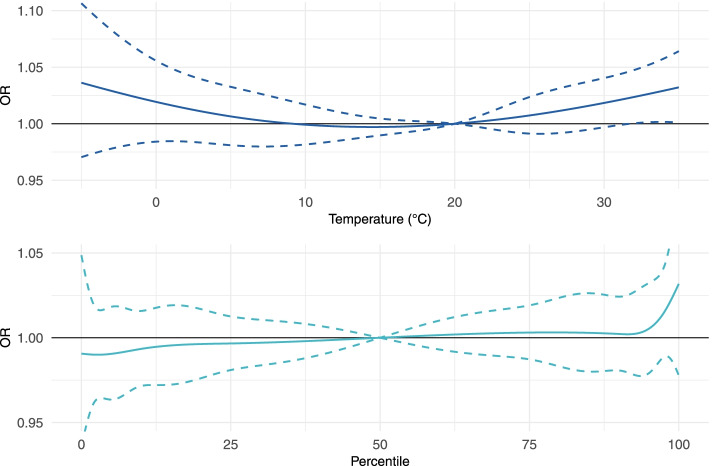
Odds ratios and 95% confidence intervals for the pooled association between continuous temperature and continuous percentile and stillbirth. All models adjusted for maternal age, LMP month, and LMP year; cases matched 1:4 to controls based on maternal race/ethnicity, education, and county

Pooled results from the absolute continuous temperature model revealed a U-shaped association between the 7-day mean temperature and stillbirth; however, there was only evidence of an increased risk at extreme temperatures and results were imprecise due to the small numbers of days observed at these temperatures (Fig. 4, Supplemental Table 5). Compared to 20 °C, an average temperature of 35 °C during the exposure window increased the risk of stillbirth [OR(CI): 1.03(1.00, 1.06)]. State specific results indicated an increased risk of stillbirth associated with extremely high temperatures in California, Florida, Kansas, and Oregon, and an increased risk of stillbirth associated with extremely low temperatures in California, Kansas, and New Jersey (Supplemental Table 5). When temperature was parameterized as a categorical variable, results showed a similar pattern to the continuous results (Supplemental Figure 2; Supplemental Table 6).

County-specific temperature distributions from 1991–2017 were used to create a relative measure of temperature by assigning county-level percentiles to the 7-day average temperature. The pooled results for continuous percentile showed no association (Fig. 4, Supplemental Table 6). There was suggestive evidence that at the highest percentiles there was an increased risk of stillbirth [OR(CI): 1.03 (0.98, 1.09), 99^th^ percentile compared to 50^th^ percentile]. Categorization of the percentiles had results comparable to the continuous estimates (Supplemental Figure 3; Supplemental Table 7).

## Discussion

In our study of 140,428 stillbirths in six states, there was some evidence that extremely elevated and sustained temperatures in the week prior to delivery increased the risk of stillbirth. There was a small but consistently elevated risk of stillbirth when a heatwave was defined using the average degrees over the threshold during the exposure window. There was also a modest increase in the odds ratio when a heatwave was defined as at least four consecutive hot days during the exposure window. Other heatwave definitions and measures of absolute and relative temperature had results that were compatible with the null.

Several studies have assessed the acute association between ambient temperature and stillbirth, but none have examined exposure to acute heatwaves prior to delivery [9]. Using a continuous measure of the mean apparent temperature for lag days 2–6, Basu et al. reported that for every 10 °C increase in the apparent temperature, there was a 10.4% increase in the excess risk of stillbirth during the warm season [11]. Similarly, in the warm season, Ha et al. found a 6% increased risk of stillbirth associated with a 1 °C increase in mean temperature during the week preceding delivery [12]. In comparison, we found that compared to 20 °C, a mean temperature of 30 °C during the exposure window (lag 0–6) increased the risk of stillbirth by 2%, which is a much smaller effect than both previously reported estimates. One potential reason we may have observed a smaller estimated association is because we implemented splines to model temperature, which allowed for nonlinearity in the effect of temperature, whereas the other studies included temperature as a linear predictor. Other differences between our study and previous studies include the full year analyses without restriction to births in the warm season, and the inclusion of over twenty years of fetal death records from six geographically diverse states. We also implemented a case–control instead of a more typical time-stratified case-crossover design, because of concerns about confounding by conception seasonality due to the strong relationship between stillbirth and gestational age. We matched cases and controls from the same location and compared the exposure of the controls at the same gestational age as the case when the case was born. This accounts for conception seasonality directly by design because spikes in conceptions affect both cases and controls.

In pooled analyses we estimated that the odds ratio associated with a 1 °C increase in the temperature over the threshold during the exposure window was 1.10 (CI: 1.04, 1.17). It is important to note that a one degree increase in this metric is extremely large, and a value for HW3 that was greater than 1 only occurred on average 1.04 days per year across counties (0.2% of days). Estimates were elevated for HW3 in all states except New Jersey, where there was no association between heatwaves and stillbirth, regardless of the definition used. This classification of a heatwave represented the most extreme definition used and incorporated both the duration and magnitude of the heatwave in the previous week, as well as county-specific thresholds. To put this into context, in Florida the average temperature in the previous week would have to be over 29.0 °C, whereas in Oregon this would only be an average degrees over 22.3 °C. Although the two temperatures differ in magnitude, they had a similar effect on the risk of stillbirth within each state [Florida: 1.28 (1.02 1.61); Oregon: 1.26 (1.02, 1.56)], providing some evidence that local extreme temperatures are important to consider instead of an overall absolute temperature measure.

An unexpected finding from our analyses was the suggestive association between extreme cold temperatures during the exposure window and stillbirth, especially in states where temperatures below 0 °C are more common. In Kansas and New Jersey, the ORs for stillbirth associated with a temperature of 0 °C, compared to 20 °C, were 1.11 (0.98, 1.25), and 1.10 (1.02, 1.19), respectively [pooled OR(CI): 1.02 (0.98, 1.06)]. This finding is similar to results by Ha et al., who reported that chronic exposure to cold temperatures during pregnancy increased the risk of stillbirth more than chronic exposure to hot temperatures; however the authors did not find evidence that acute exposure to cold temperatures increased the risk of stillbirth [12]. When temperatures were classified using the county-specific percentiles, the elevated risk of stillbirth was still isolated to the states with the coldest absolute temperatures. This differs from the results for extreme hot temperatures, where both high absolute and relative temperature were associated with an increased risk of stillbirth. The null association between the lowest percentiles and stillbirth may provide evidence that the risk of stillbirth associated with cold temperatures is more dependent on absolute temperature, rather than relative temperature.

Although race/ethnicity is an unlikely confounder in this context due to purely temporal contrasts of exposure, we examined race/ethnicity as a possible effect modifier and observed some evidence that there was an increased risk of stillbirth among non-Hispanic black mothers, which is consistent with previous studies examining racial differences in the association between environmental exposures and birth outcomes [20, 22, 23]. Additionally, state-specific results did have some patterns of elevated risk within certain maternal race/ethnicity categories. There was an increased risk of stillbirth associated with both HW2 and HW3 among white, non-Hispanic women in Kansas and among black, non-Hispanic women in Florida. It is unclear why these particular groups would have an elevated risk in these states, but it is possible that race/ethnicity is serving as a proxy for unmeasured sociodemographic factors [24]. One limitation to our analysis was missing information on maternal race on the Oregon fetal death records, and therefore we could not match on maternal race or include Oregon in stratified analyses.

We hypothesized that stillbirths after 28 weeks gestation would be more sensitive to the acute effects of extreme temperatures, but despite the potential etiologic differences between early and late stillbirths, we did not find evidence that the timing of the stillbirth in pooled or state-specific results changed the conclusions. Across states, there were differences in the proportion of stillbirths that were delivered before 28 weeks compared to the proportion that were delivered after 28 weeks. However, when looking at stillbirths as a percent of all pregnancies (Supplemental Table 1), the proportion of late stillbirths was similar across states (~ 0.25% of all pregnancies) whereas the proportion of early stillbirths varied across states. We believe this difference is due to each state’s required reporting of fetal deaths, and their varied ability to document very early stillbirths (i.e., those around 20 weeks gestation).

Acute risk factors for stillbirth are difficult to investigate because the timing of the fetal death is unknown, and instead studies must rely on the date of delivery. Because of this, there may have been misclassification of the exposure, using the week before delivery instead of the week before fetal death. Most fetal deaths occur within two days of delivery [25], meaning that at least five days prior to death occurred during the exposure window, which is a reasonable proxy for acute exposure. A second limitation to this study is that it relied on the accuracy of data on the fetal death records and birth records, which may vary across states and over time. However, studies have reported that the least accurate information on vital records relate to maternal comorbidities and prenatal care, which were not used in this study [26, 27]. Finally, this study is that the aggregated county-level temperature estimates may not reflect an individuals’ true exposure due to temperature differences within a county or mitigation factors individuals use when it is hot outside (e.g., staying inside, using air conditioning, hydrating, etc.).

This is the largest study to date that has examined the association between acute heatwaves and stillbirth. Even with this extremely large sample, estimates were often imprecise due to both heatwaves and stillbirths being rare events, but associations were estimated with more precision than previous studies. We used a geographically diverse sample to examine multiple heatwave metrics that took into account county-level thresholds and local acclimatization; results varied by how heatwaves and temperatures were operationalized. We found evidence that only the most extreme heatwave definitions and continuous temperature metrics were associated with a modest increase in the risk of stillbirth. These results are important in the context of increasing global temperatures and highlight a potential risk-factor for stillbirth that is likely to become more common in the future. Although heatwaves are not a modifiable risk factor, healthcare providers should be aware of the potential effects of extreme temperatures and should counsel patients to take preventive measures during periods of extreme heat. Future studies are needed to support our findings and should aim to assess the association between acute heatwaves and stillbirth using a variety of heatwave and temperature metrics in large, geographically diverse populations.

## Supplementary Information

**Additional file 1: Supplemental Figure 1.** County-level 7-day mean temperature distribution by state, 1991-2017. **Supplemental Table 1.** Rate of stillbirth across states. **Supplemental Table 2.** Odds ratios and 95% confidence intervals for the association between heatwaves and stillbirth in California, Florida, Georgia, Kansas, New Jersey, and Oregon. **Supplemental Table 3.** Odds ratios and 95% confidence intervals for the associations between heatwaves and stillbirth by maternal race, by state. **Supplemental Table 4.** Odds ratios and 95% confidence intervals for the association between heatwaves and stillbirth by the timing of the stillbirth, by state. **Supplemental Table 5.** Select estimates for continuous temperature model, reference 20°C. **Supplemental Table 6.** Select estimates for continuous percentile model, reference 50%. **Supplemental Figure 2.** Comparison of continuous temperature model versus categorical temperature model, pooled results. **Supplemental Figure 3.** Comparison of continuous percentile model versus categorical temperature model, pooled results. **Supplemental Table 7.** Odds ratios and 95% confidence intervals for categorical temperature model. **Supplemental Table 8.** Odds ratios and 95% confidence intervals for categorical percentile model.

## Data Availability

The data that support the findings of this study are available from individual states but restrictions apply to the availability of these data, which were used under license for the current study, and so are not publicly available.
