# Supplementary material for: Acute association between heatwaves and stillbirth in six US states
Source: Environ Health. 2022 Jun 16;21:59. doi: 10.1186/s12940-022-00870-y (PMC9202158; doi:10.1186/s12940-022-00870-y)
Supplement: Supplementary file 1 — Additional file 1: Supplemental Figure 1. County-level 7-day mean temperature distribution by state, 1991-2017. Supplemental Table 1. Rate of stillbirth across states. Supplemental Table 2. Odds ratios and 95% confidence intervals for the association between heatwaves and stillbirth in California, Florida, Georgia, Kansas, New Jersey, and Oregon. Supplemental Table 3. Odds ratios and 95% confidence intervals for the associations between heatwaves and stillbirth by maternal race, by state. Supplemental Table 4. Odds ratios and 95% confidence intervals for the association between heatwaves and stillbirth by the timing of the stillbirth, by state. Supplemental Table 5. Select estimates for continuous temperature model, reference 20°C. Supplemental Table 6. Select estimates for continuous percentile model, reference 50%. Supplemental Figure 2. Comparison of continuous temperature model versus categorical temperature model, pooled results. Supplemental Figure 3. Comparison of continuous percentile model versus categorical temperature model, pooled results. Supplemental Table 7. Odds ratios and 95% confidence intervals for categorical temperature model. Supplemental Table 8. Odds ratios and 95% confidence intervals for categorical percentile model. [file 12940_2022_870_MOESM1_ESM.docx]

| Supplemental Figure 1. County-level 7-day mean temperature distribution by state, 1991-2017 |
| --- |
| 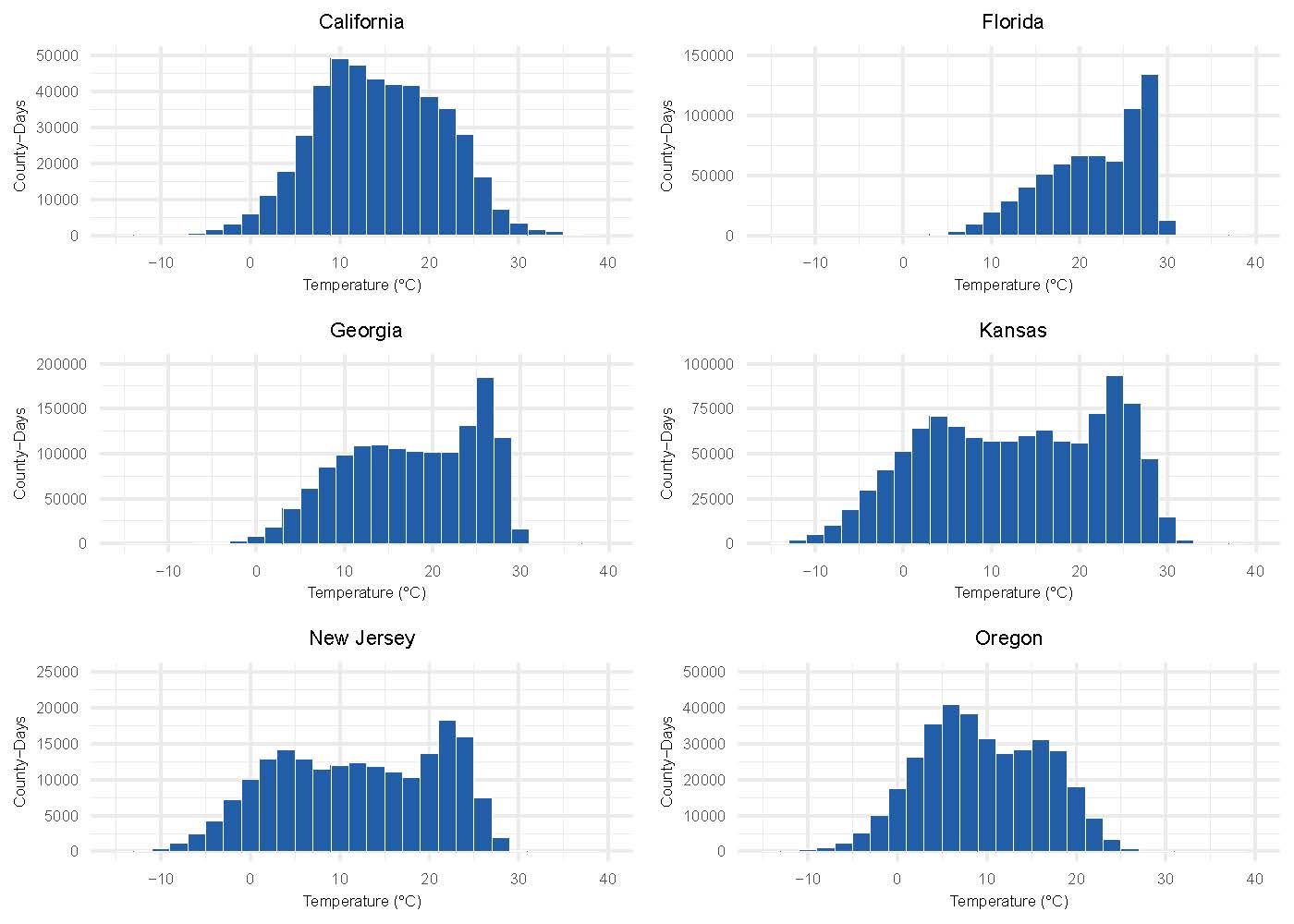 |
|  |

| Supplemental Table 1. Rate of stillbirth across states | | | | | | | | | | | | | | | | | | | |
| --- | --- | --- | --- | --- | --- | --- | --- | --- | --- | --- | --- | --- | --- | --- | --- | --- | --- | --- | --- |
|  | California | | | Florida | | | Georgia | | | Kansas | | | New Jersey | | | Oregon | | | |
|  | Overall | Early (<28 weeks) | Late (≥28 weeks) | Overall | Early (<28 weeks) | Late (≥28 weeks) | Overall | Early (<28 weeks) | Late (≥28 weeks) | Overall | Early (<28 weeks) | Late (≥28 weeks) | Overall | Early (<28 weeks) | Late (≥28 weeks) | Overall | Early (<28 weeks) | Late (≥28 weeks) |  |
| 1991 |  |  |  | 0.79% | 0.36% | 0.43% |  |  |  | 0.51% | 0.21% | 0.30% | 0.64% | 0.33% | 0.31% | 0.57% | 0.27% | 0.30% |  |
| 1992 |  |  |  | 0.76% | 0.36% | 0.40% |  |  |  | 0.54% | 0.23% | 0.32% | 0.58% | 0.30% | 0.28% | 0.54% | 0.22% | 0.32% |  |
| 1993 |  |  |  | 0.74% | 0.37% | 0.36% |  |  |  | 0.59% | 0.21% | 0.38% | 0.57% | 0.29% | 0.28% | 0.48% | 0.22% | 0.26% |  |
| 1994 |  |  |  | 0.80% | 0.43% | 0.37% | 1.08% | 0.66% | 0.42% | 0.59% | 0.19% | 0.39% | 0.57% | 0.30% | 0.28% | 0.52% | 0.22% | 0.29% |  |
| 1995 |  |  |  | 0.81% | 0.41% | 0.40% | 1.23% | 0.75% | 0.48% | 0.46% | 0.18% | 0.28% | 0.59% | 0.34% | 0.26% | 0.52% | 0.21% | 0.31% |  |
| 1996 | 0.59% | 0.22% | 0.37% | 0.78% | 0.42% | 0.36% | 1.02% | 0.65% | 0.37% | 0.47% | 0.17% | 0.30% | 0.55% | 0.31% | 0.24% | 0.55% | 0.22% | 0.32% |  |
| 1997 | 0.55% | 0.20% | 0.35% | 0.79% | 0.43% | 0.36% | 1.04% | 0.65% | 0.38% | 0.52% | 0.21% | 0.31% | 0.56% | 0.31% | 0.25% | 0.50% | 0.19% | 0.31% |  |
| 1998 | 0.57% | 0.21% | 0.37% | 0.79% | 0.44% | 0.35% | 1.00% | 0.63% | 0.37% | 0.51% | 0.17% | 0.33% | 0.55% | 0.31% | 0.24% | 0.48% | 0.22% | 0.26% |  |
| 1999 | 0.55% | 0.21% | 0.34% | 0.81% | 0.45% | 0.37% | 0.99% | 0.62% | 0.36% | 0.43% | 0.15% | 0.28% | 0.72% | 0.42% | 0.30% | 0.49% | 0.24% | 0.25% |  |
| 2000 | 0.55% | 0.21% | 0.34% | 0.83% | 0.48% | 0.35% | 0.93% | 0.61% | 0.31% | 0.43% | 0.15% | 0.28% | 0.65% | 0.38% | 0.27% | 0.44% | 0.20% | 0.24% |  |
| 2001 | 0.54% | 0.21% | 0.33% | 0.83% | 0.48% | 0.35% | 1.00% | 0.65% | 0.35% | 0.50% | 0.19% | 0.32% | 0.72% | 0.41% | 0.31% | 0.49% | 0.20% | 0.29% |  |
| 2002 | 0.52% | 0.20% | 0.32% | 0.79% | 0.45% | 0.34% | 1.06% | 0.71% | 0.35% | 0.35% | 0.12% | 0.23% | 0.65% | 0.39% | 0.26% | 0.52% | 0.22% | 0.30% |  |
| 2003 | 0.52% | 0.20% | 0.32% | 0.78% | 0.45% | 0.34% | 1.00% | 0.64% | 0.36% | 0.50% | 0.21% | 0.30% | 0.69% | 0.41% | 0.29% | 0.41% | 0.15% | 0.26% |  |
| 2004 | 0.51% | 0.19% | 0.32% | 0.81% | 0.48% | 0.33% | 1.01% | 0.67% | 0.34% | 0.44% | 0.17% | 0.28% | 0.67% | 0.40% | 0.27% | 0.42% | 0.15% | 0.27% |  |
| 2005 | 0.52% | 0.21% | 0.31% | 0.74% | 0.43% | 0.31% | 0.96% | 0.61% | 0.35% | 0.49% | 0.16% | 0.33% | 0.65% | 0.42% | 0.23% | 0.42% | 0.22% | 0.20% |  |
| 2006 | 0.51% | 0.20% | 0.31% | 0.76% | 0.43% | 0.33% | 1.01% | 0.66% | 0.35% | 0.42% | 0.12% | 0.29% | 0.70% | 0.44% | 0.26% | 0.44% | 0.19% | 0.25% |  |
| 2007 | 0.49% | 0.24% | 0.25% | 0.79% | 0.44% | 0.35% | 0.89% | 0.58% | 0.31% | 0.42% | 0.15% | 0.28% | 0.68% | 0.42% | 0.26% | 0.45% | 0.20% | 0.25% |  |
| 2008 | 0.49% | 0.24% | 0.25% | 0.75% | 0.41% | 0.34% | 0.74% | 0.43% | 0.30% | 0.42% | 0.14% | 0.27% | 0.64% | 0.41% | 0.23% | 0.42% | 0.16% | 0.26% |  |
| 2009 | 0.48% | 0.23% | 0.25% | 0.73% | 0.40% | 0.33% | 0.76% | 0.46% | 0.30% | 0.48% | 0.18% | 0.30% | 0.56% | 0.34% | 0.22% | 0.44% | 0.13% | 0.32% |  |
| 2010 | 0.48% | 0.24% | 0.24% | 0.74% | 0.41% | 0.32% | 0.73% | 0.43% | 0.30% | 0.40% | 0.14% | 0.26% | 0.67% | 0.40% | 0.28% | 0.40% | 0.15% | 0.24% |  |
| 2011 | 0.47% | 0.22% | 0.25% | 0.75% | 0.42% | 0.33% | 0.82% | 0.49% | 0.33% | 0.45% | 0.17% | 0.28% | 0.56% | 0.35% | 0.21% | 0.39% | 0.14% | 0.25% |  |
| 2012 | 0.47% | 0.23% | 0.24% | 0.74% | 0.42% | 0.33% | 0.76% | 0.44% | 0.32% | 0.45% | 0.13% | 0.32% | 0.60% | 0.37% | 0.24% | 0.42% | 0.15% | 0.27% |  |
| 2013 | 0.49% | 0.23% | 0.26% | 0.73% | 0.42% | 0.31% | 0.81% | 0.49% | 0.32% | 0.42% | 0.14% | 0.28% | 0.61% | 0.36% | 0.25% | 0.41% | 0.15% | 0.26% |  |
| 2014 | 0.49% | 0.24% | 0.26% | 0.74% | 0.42% | 0.32% | 0.78% | 0.46% | 0.32% | 0.47% | 0.22% | 0.25% | 0.54% | 0.33% | 0.22% | 0.41% | 0.13% | 0.28% |  |
| 2015 | 0.48% | 0.23% | 0.25% | 0.71% | 0.40% | 0.31% | 0.84% | 0.49% | 0.35% | 0.56% | 0.32% | 0.25% | 0.50% | 0.29% | 0.20% | 0.39% | 0.15% | 0.24% |  |
| 2016 | 0.49% | 0.24% | 0.25% | 0.71% | 0.39% | 0.31% | 0.80% | 0.45% | 0.36% | 0.64% | 0.29% | 0.35% |  |  |  | 0.40% | 0.16% | 0.24% |  |
| 2017 | 0.48% | 0.23% | 0.25% | 0.71% | 0.38% | 0.33% | 0.78% | 0.45% | 0.33% | 0.48% | 0.25% | 0.23% |  |  |  | 0.42% | 0.14% | 0.28% |  |
| 2018 |  |  |  |  |  |  |  |  |  | 0.51% | 0.28% | 0.24% |  |  |  |  |  |  |  |

| Supplemental Table 2. Odds ratios and 95% confidence intervals for the association between heatwaves and stillbirth in California, Florida, Georgia, Kansas, New Jersey, and Oregon | | | | | | | |
| --- | --- | --- | --- | --- | --- | --- | --- |
|  | HW1 | | | HW2 | | | HW3 |
|  | 1 Day | 2 Days | 3+ Days | ≥2 Consecutive | ≥3 Consecutive | ≥4 Consecutive |  |
| California | 1.02 (0.97, 1.06) | 1.01 (0.96, 1.06) | 0.98 (0.93, 1.02) | 1.00 (0.97, 1.02) | 0.99 (0.96, 1.02) | 1.03 (0.99, 1.07) | 1.07 (0.99, 1.16) |
| Florida | 0.99 (0.94, 1.05) | 0.95 (0.88, 1.02) | 1.08 (1.02, 1.15) | 1.03 (1.00, 1.06) | 1.04 (1.00, 1.08) | 1.05 (1.00, 1.10) | 1.28 (1.02, 1.61) |
| Georgia | 1.07 (1.00, 1.16) | 0.92 (0.85, 1.00) | 1.00 (0.93, 1.07) | 0.98 (0.95, 1.02) | 0.99 (0.95, 1.04) | 1.01 (0.96, 1.07) | 1.10 (0.95, 1.26) |
| Kansas | 0.88 (0.74, 1.05) | 1.16 (0.96, 1.40) | 1.07 (0.91, 1.25) | 1.11 (1.02, 1.20) | 1.10 (1.00, 1.22) | 1.13 (0.99, 1.29) | 1.22 (0.96, 1.54) |
| New Jersey | 1.01 (0.92, 1.10) | 0.97 (0.88, 1.08) | 0.97 (0.88, 1.07) | 0.97 (0.93, 1.02) | 0.97 (0.91, 1.04) | 0.98 (0.89, 1.07) | 1.01 (0.83, 1.22) |
| Oregon | 0.88 (0.76, 1.03) | 1.08 (0.92, 1.27) | 1.11 (0.95, 1.29) | 1.08 (1.01, 1.16) | 1.09 (0.99, 1.20) | 1.12 (0.97, 1.29) | 1.26 (1.02, 1.56) |
| **Pooled** | 1.01 (0.98, 1.04) | 0.98 (0.95, 1.02) | 1.01 (0.98, 1.04) | 1.00 (0.99, 1.02) | 1.00 (0.99, 1.03) | 1.03 (1.01, 1.06) | 1.10 (1.04, 1.17) |
| All models adjusted for maternal age, LMP month, and LMP year; cases matched 1:4 to controls based on maternal race, education, and county | | | | | | | |

| Supplemental Table 3. Odds ratios and 95% confidence intervals for the associations between heatwaves and stillbirth by maternal race, by state | | | | | | | | |
| --- | --- | --- | --- | --- | --- | --- | --- | --- |
|  |  | HW1 | | | HW2 | | | HW3 |
|  |  | 1 Day | 2 Days | 3+ Days | ≥2 Consecutive | ≥3 Consecutive | ≥4 Consecutive |  |
| White, NH | California | 1.09 (0.99, 1.19) | 1.00 (0.90, 1.11) | 0.92 (0.84, 1.01) | 0.98 (0.93, 1.02) | 0.96 (0.91, 1.02) | 1.03 (0.95, 1.11) | 1.12 (0.96, 1.29) |
|  | Florida | 1.04 (0.95, 1.14) | 0.94 (0.84, 1.05) | 1.03 (0.94, 1.13) | 1.00 (0.95, 1.05) | 1.00 (0.94, 1.07) | 1.00 (0.92, 1.09) | 1.11 (0.78, 1.57) |
|  | Georgia | 1.97 (1.74, 2.24) | 0.85 (0.73, 0.98) | 1.07 (0.95, 1.20) | 0.97 (0.92, 1.03) | 1.01 (0.94, 1.08) | 1.05 (0.96, 1.16) | 1.27 (1.00, 1.60) |
|  | Kansas | 0.79 (0.63, 0.99) | 1.21 (0.96, 1.54) | 1.13 (0.92, 1.38) | 1.14 (1.03, 1.25) | 1.14 (1.00, 1.29) | 1.18 (1.01, 1.38) | 1.43 (1.06, 1.92) |
|  | New Jersey | 1.07 (0.94, 1.24) | 0.90 (0.76, 1.07) | 0.92 (0.78, 1.08) | 0.92 (0.85, 0.99) | 0.94 (0.84, 1.04) | 0.99 (0.85, 1.15) | 1.13 (0.82, 1.55) |
|  | **Pooled** | 1.04 (0.99, 1.10) | 0.95 (0.90, 1.01) | 1.00 (0.95, 1.05) | 0.99 (0.96, 1.01) | 0.99 (0.96, 1.03) | 1.03 (0.99, 1.08) | 1.18 (1.06, 1.30) |
| Black, NH | California | 0.98 (0.85, 1.12) | 1.12 (0.97, 1.30) | 0.92 (0.80, 1.05) | 1.01 (0.94, 1.07) | 0.94 (0.87, 1.03) | 1.04 (0.93, 1.17) | 0.97 (0.76, 1.25) |
|  | Florida | 0.96 (0.87, 1.07) | 0.97 (0.86, 1.10) | 1.17 (1.06, 1.29) | 1.09 (1.04, 1.15) | 1.13 (1.06, 1.21) | 1.13 (1.04, 1.23) | 1.75 (1.21, 2.52) |
|  | Georgia | 1.04 (0.93, 1.15) | 1.00 (0.90, 1.12) | 0.96 (0.88, 1.06) | 1.00 (0.95, 1.04) | 0.98 (0.93, 1.04) | 0.98 (0.90, 1.06) | 0.95 (0.77, 1.17) |
|  | Kansas | 0.77 (0.44, 1.34) | 1.33 (0.79, 2.24) | 1.07 (0.69, 1.68) | 1.09 (0.88, 1.35) | 1.15 (0.87, 1.51) | 1.30 (0.91, 1.85) | 1.12 (0.55, 2.25) |
|  | New Jersey | 0.95 (0.81, 1.12) | 0.93 (0.77, 1.12) | 1.04 (0.88, 1.23) | 0.98 (0.90, 1.06) | 1.00 (0.89, 1.11) | 1.01 (0.87, 1.18) | 0.97 (0.70, 1.34) |
|  | **Pooled** | 0.99 (0.93, 1.04) | 1.01 (0.95, 1.08) | 1.03 (0.97, 1.09) | 1.02 (1.00, 1.05) | 1.02 (0.99, 1.06) | 1.05 (1.00, 1.10) | 1.04 (0.91, 1.19) |
| Hispanic | California | 1.02 (0.96, 1.09) | 1.01 (0.94, 1.09) | 1.01 (0.95, 1.08) | 1.03 (1.00, 1.06) | 1.03 (0.99, 1.08) | 1.03 (0.97, 1.09) | 1.03 (0.92, 1.15) |
|  | Florida | 0.97 (0.86, 1.08) | 0.92 (0.80, 1.06) | 1.06 (0.95, 1.20) | 0.97 (0.92, 1.03) | 1.00 (0.92, 1.08) | 1.01 (0.91, 1.12) | 1.03 (0.59, 1.79) |
|  | Georgia | 1.21 (0.95, 1.55) | 0.98 (0.73, 1.32) | 0.85 (0.67, 1.08) | 0.96 (0.30, 3.05) | 0.93 (0.21, 4.16) | 0.86 (0.70, 1.06) | 1.08 (0.66, 1.74) |
|  | Kansas | 1.29 (0.89, 1.86) | 0.99 (0.63, 1.55) | 0.93 (0.65, 1.33) | 1.09 (0.92, 1.30) | 1.04 (0.82, 1.31) | 0.88 (0.61, 1.27) | 0.90 (0.53, 1.52) |
|  | New Jersey | 1.04 (0.86, 1.25) | 0.98 (0.79, 1.22) | 0.96 (0.78, 1.17) | 0.96 (0.87, 1.06) | 0.96 (0.84, 1.10) | 0.90 (0.73, 1.10) | 0.92 (0.61, 1.41) |
|  | **Pooled** | 1.02 (0.96, 1.07) | 0.99 (0.93, 1.05) | 1.00 (0.95, 1.05) | 1.00 (0.98, 1.03) | 1.00 (0.97, 1.04) | 1.00 (0.96, 1.05) | 1.04 (0.93, 1.15) |
| Other | California | 0.94 (0.82, 1.07) | 1.02 (0.87, 1.18) | 0.97 (0.84, 1.12) | 0.95 (0.89, 1.02) | 0.95 (0.86, 1.04) | 1.02 (0.89, 1.16) | 1.13 (0.91, 1.42) |
|  | Florida | 0.87 (0.56, 1.36) | 1.23 (0.73, 2.09) | 1.05 (0.68, 1.62) | 1.17 (0.93, 1.45) | 1.00 (0.75, 1.33) | 0.91 (0.60, 1.37) | 0.90 (0.22, 3.77) |
|  | Georgia | 0.81 (0.48, 1.37) | 0.68 (0.37, 1.24) | 1.45 (0.98, 2.14) | 1.00 (0.82, 1.21) | 1.15 (0.92, 1.43) | 1.39 (1.09, 1.78) | 1.61 (0.92, 2.82) |
|  | Kansas | 1.40 (0.51, 3.84) | 0.74 (0.21, 2.62) | 0.51 (0.15, 1.67) | 0.58 (0.33, 1.02) | 0.57 (0.27, 1.21) | 0.60 (0.21, 1.73) | 0.42 (0.05, 3.20) |
|  | New Jersey | 0.89 (0.65, 1.22) | 1.63 (1.13, 2.35) | 0.95 (0.66, 1.36) | 1.28 (1.08, 1.53) | 2.55 (2.00, 3.26) | 0.95 (0.66, 1.35) | 0.84 (0.40, 1.77) |
|  | **Pooled** | 0.92 (0.82, 1.04) | 1.06 (0.93, 1.20) | 0.99 (0.88, 1.12) | 1.00 (0.94, 1.06) | 0.98 (0.91, 1.06) | 1.06 (0.95, 1.18) | 1.14 (0.93, 1.42) |
| All models adjusted for maternal age, LMP month, and LMP year; cases matched 1:4 to controls based on maternal race, education, and county | | | | | | | | |

| Supplemental Table 4. Odds ratios and 95% confidence intervals for the association between heatwaves and stillbirth by the timing of the stillbirth, by state | | | | | | | | |
| --- | --- | --- | --- | --- | --- | --- | --- | --- |
|  |  | HW1 | | | HW2 | | | HW3 |
|  |  | 1 Day | 2 Days | 3+ Days | ≥2 Consecutive | ≥3 Consecutive | ≥4 Consecutive |  |
| Early (<28) | California | 1.05 (0.98, 1.12) | 0.97 (0.90, 1.05) | 0.98 (0.91, 1.05) | 0.99 (0.95, 1.02) | 0.98 (0.94, 1.03) | 1.04 (0.98, 1.11) | 1.05 (0.93, 1.19) |
|  | Florida | 1.00 (0.92, 1.08) | 0.96 (0.87, 1.06) | 1.06 (0.98, 1.15) | 1.02 (0.98, 1.07) | 1.03 (0.98, 1.09) | 1.03 (0.96, 1.11) | 1.26 (0.91, 1.73) |
|  | Georgia | 1.08 (0.98, 1.19) | 0.93 (0.84, 1.03) | 1.00 (0.92, 1.09) | 0.99 (0.95, 1.03) | 1.00 (0.95, 1.06) | 1.03 (0.96, 1.11) | 1.10 (0.92, 1.33) |
|  | Kansas | 0.68 (0.50, 0.93) | 1.22 (0.89, 1.69) | 1.22 (0.93, 1.61) | 1.12 (0.98, 1.29) | 1.16 (0.98, 1.38) | 1.15 (0.91, 1.44) | 1.00 (0.62, 1.61) |
|  | Oregon | 0.72 (0.70, 0.73) | 1.19 (0.92, 1.54) | 1.15 (0.89, 1.47) | 1.10 (0.98, 1.24) | 1.06 (0.90, 1.25) | 1.16 (0.91, 1.48) | 1.18 (0.79, 1.75) |
|  | New Jersey | 1.62 (0.55, 4.76) | 0.64 (0.22, 1.82) | 0.57 (0.12, 2.78) | 0.64 (0.38, 1.09) | 0.59 (0.21, 1.63) | N/A | N/A |
|  | **Pooled** | 1.01 (0.97, 1.06) | 0.97 (0.92, 1.02) | 1.02 (0.98, 1.07) | 1.00 (0.98, 1.03) | 1.01 (0.98, 1.04) | 1.04 (1.00, 1.08) | 1.08 (0.99, 1.19) |
| Late (≥28) | California | 0.99 (0.93, 1.05) | 1.05 (0.98, 1.12) | 0.97 (0.91, 1.03) | 1.00 (0.97, 1.03) | 0.99 (0.95, 1.03) | 1.02 (0.96, 1.07) | 1.08 (0.98, 1.20) |
|  | Florida | 0.98 (0.90, 1.07) | 0.93 (0.84, 1.03) | 1.10 (1.01, 1.19) | 1.02 (0.97, 1.06) | 1.04 (0.99, 1.10) | 1.05 (0.98, 1.13) | 1.27 (0.92, 1.76) |
|  | Georgia | 1.06 (0.94, 1.20) | 0.92 (0.80, 1.06) | 0.98 (0.88, 1.09) | 0.97 (0.91, 1.02) | 0.97 (0.90, 1.03) | 0.98 (0.89, 1.07) | 1.08 (0.86, 1.35) |
|  | Kansas | 0.99 (0.80, 1.22) | 1.13 (0.89, 1.43) | 0.97 (0.79, 1.18) | 1.07 (0.97, 1.19) | 1.04 (0.92, 1.18) | 1.09 (0.93, 1.29) | 1.25 (0.95, 1.66) |
|  | Oregon | 0.98 (0.82, 1.19) | 1.00 (0.81, 1.23) | 1.08 (0.89, 1.30) | 1.05 (0.96, 1.15) | 1.09 (0.96, 1.23) | 1.07 (0.89, 1.28) | 1.25 (0.96, 1.61) |
|  | New Jersey | 1.01 (0.88, 1.15) | 1.01 (0.87, 1.18) | 0.96 (0.83, 1.12) | 0.99 (0.92, 1.06) | 0.99 (0.90, 1.10) | 1.00 (0.88, 1.15) | 1.02 (0.75, 1.38) |
|  | **Pooled** | 1.00 (0.95, 1.04) | 1.00 (0.95, 1.05) | 1.01 (0.97, 1.05) | 1.00 (0.98, 1.03) | 1.01 (0.98, 1.03) | 1.02 (0.99, 1.06) | 1.12 (1.04, 1.22) |
| All models adjusted for maternal age, LMP month, and LMP year; cases matched 1:4 to controls based on maternal race, education, and county | | | | | | | | |

| Supplemental Table 5. Select estimates for continuous temperature model, reference 20°C | | | | | | |
| --- | --- | --- | --- | --- | --- | --- |
|  | -5°C | 0°C | 10°C | 20°C | 30°C | 35°C |
| California | 1.07 (0.87, 1.31) | 1.02 (0.93, 1.12) | 0.99 (0.96, 1.02) | REF | 1.03 (0.98, 1.07) | 1.07 (0.97, 1.19) |
| Florida | 1.03 (0.76, 1.39) | 1.04 (0.89, 1.21) | 1.01 (0.96, 1.07) | REF | 1.05 (1.00, 1.11) | 1.05 (1.01, 1.10) |
| Georgia | 0.99 (0.89, 1.10) | 0.97 (0.92, 1.03) | 0.98 (0.94, 1.02) | REF | 0.99 (0.95, 1.04) | 1.01 (0.94, 1.08) |
| Kansas | 1.17 (0.96, 1.42) | 1.11 (0.98, 1.25) | 1.04 (0.96, 1.13) | REF | 1.03 (0.94, 1.14) | 1.18 (0.98, 1.43) |
| New Jersey | 1.06 (0.93, 1.19) | 1.10 (1.02, 1.19) | 1.07 (1.02, 1.12) | REF | 0.98 (0.91, 1.06) | 0.91 (0.78, 1.06) |
| Oregon | 0.92 (0.64, 1.32) | 0.95 (0.77, 1.16) | 0.95 (0.86, 1.05) | REF | 1.19 (0.91, 1.54) | 1.24 (0.70, 2.20) |
| **Pooled** | 1.04 (0.97, 1.11) | 1.02 (0.98, 1.06) | 1 (0.98, 1.02) | REF | 1.02 (1.00, 1.04) | 1.03 (1.00, 1.06) |
| All models adjusted for maternal age, LMP month, and LMP year; cases matched 1:4 to controls based on maternal race, education, and county Estimates represent extrapolations from models, and not all temperatures were observed in all states | | | | | | |

| Supplemental Table 6. Select estimates for continuous percentile model, reference 50% | | | | | | | | | |  |
| --- | --- | --- | --- | --- | --- | --- | --- | --- | --- | --- |
|  | 1% | 2.5% | 10% | 20% | 50% | 80% | 90% | 97.5% | 99% | |
| California | 1.01 (0.95, 1.08) | 1.00 (0.95, 1.04) | 1.00 (0.97, 1.04) | 1.01 (0.98, 1.05) | REF | 1.02 (0.99, 1.06) | 1.02 (0.98, 1.05) | 1.01 (0.97, 1.05) | 1.02 (0.94, 1.12) | |
| Florida | 0.97 (0.89, 1.05) | 0.97 (0.92, 1.02) | 0.96 (0.92, 1.01) | 0.96 (0.93, 1.00) | REF | 1.00 (0.96, 1.04) | 0.99 (0.95, 1.03) | 1.04 (0.99, 1.10) | 1.08 (1.00, 1.17) | |
| Georgia | 0.92 (0.84, 1.01) | 0.97 (0.91, 1.03) | 0.98 (0.93, 1.03) | 0.95 (0.91, 1.00) | REF | 0.98 (0.94, 1.03) | 1.00 (0.95, 1.05) | 1.00 (0.94, 1.06) | 0.96 (0.85, 1.08) | |
| Kansas | 1.16 (0.93, 1.43) | 1.10 (0.95, 1.27) | 1.04 (0.92, 1.18) | 1.05 (0.94, 1.18) | REF | 0.96 (0.86, 1.07) | 1.01 (0.90, 1.14) | 1.05 (0.92, 1.21) | 1.00 (0.74, 1.34) | |
| New Jersey | 1.04 (0.92, 1.19) | 1.03 (0.94, 1.12) | 1.05 (0.97, 1.13) | 1.08 (1.01, 1.15) | REF | 0.96 (0.90, 1.03) | 0.96 (0.89, 1.03) | 0.92 (0.84, 1.01) | 0.90 (0.75, 1.08) | |
| Oregon | 1.04 (0.84, 1.28) | 0.96 (0.83, 1.11) | 1.01 (0.90, 1.13) | 1.06 (0.96, 1.18) | REF | 1.07 (0.96, 1.18) | 1.05 (0.94, 1.17) | 1.17 (1.02, 1.33) | 1.37 (1.04, 1.80) | |
| **Pooled** | 0.99 (0.95, 1.03) | 0.99 (0.96, 1.02) | 0.99 (0.97, 1.02) | 1.00 (0.98, 1.02) | REF | 1.00 (0.98, 1.02) | 1.00 (0.98, 1.02) | 1.01 (0.99, 1.04) | 1.02 (0.99, 1.06) | |

All models adjusted for maternal age, LMP month, and LMP year; cases matched 1:4 to controls based on maternal race, education, and county

| Supplemental Figure 2. Comparison of continuous temperature model versus categorical temperature model, pooled results |
| --- |
| 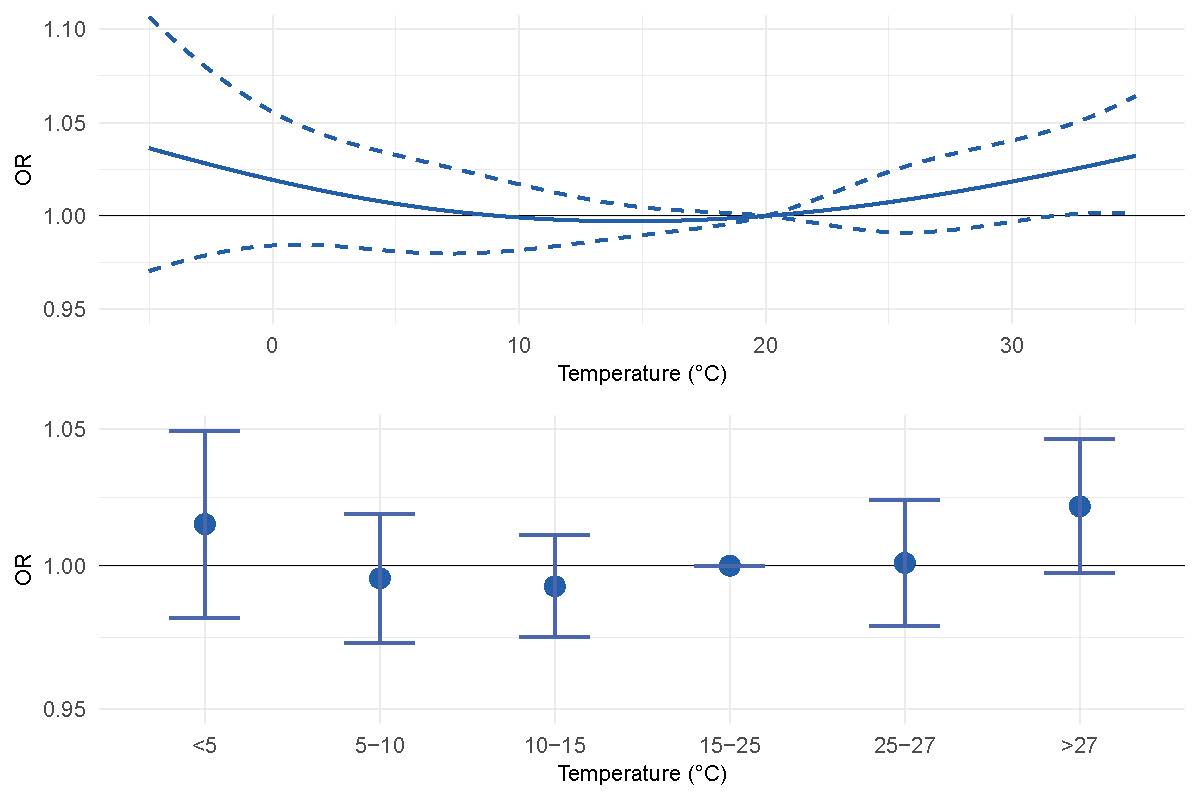 |
|  |
| All models adjusted for maternal age, LMP month, and LMP year; cases matched 1:4 to controls based on maternal race, education, and county |
|  |
|  |

| Supplemental Figure 3. Comparison of continuous percentile model versus categorical temperature model, pooled results |
| --- |
| 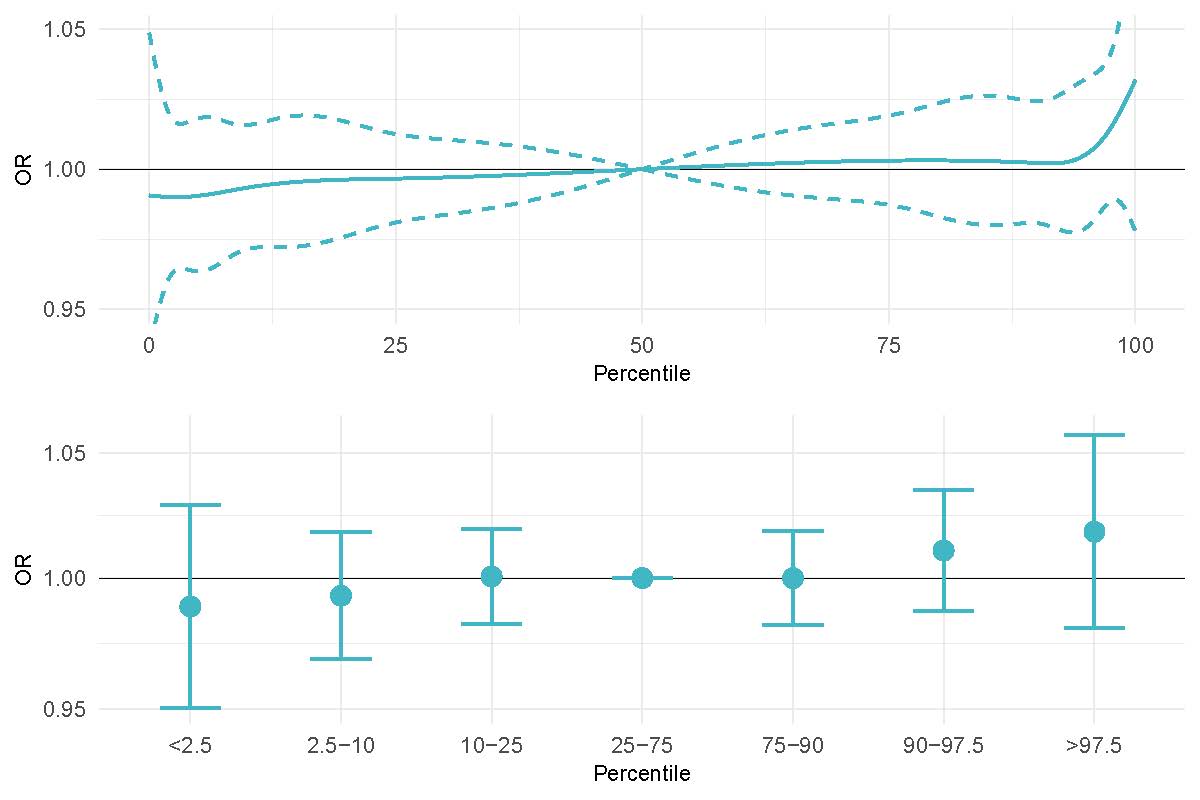 |
|  |
| All models adjusted for maternal age, LMP month, and LMP year; cases matched 1:4 to controls based on maternal race, education, and county |

| Supplemental Table 7. Odds ratios and 95% confidence intervals for categorical temperature model | | | | | | |
| --- | --- | --- | --- | --- | --- | --- |
|  | <5 °C | 5-10 °C | 10-15 °C | 15-25 °C | 25-27 °C | >27 °C |
| California | 0.98 (0.90, 1.08) | 0.98 (0.95, 1.01) | 1.01 (0.98, 1.03) | REF | 1.01 (0.97, 1.06) | 1.03 (0.98, 1.09) |
| Florida | 0.64 (0.61, 0.67) | 1.06 (0.96, 1.18) | 0.97 (0.92, 1.02) | REF | 1.02 (0.98, 1.06) | 1.02 (0.99, 1.06) |
| Georgia | 0.99 (0.93, 1.05) | 0.97 (0.92, 1.01) | 0.96 (0.93, 1.00) | REF | 0.96 (0.92, 1.00) | 1.01 (0.96, 1.07) |
| Kansas | 1.07 (0.96, 1.19) | 1.04 (0.93, 1.16) | 0.99 (0.89, 1.11) | REF | 1.05 (0.92, 1.19) | 1.05 (0.92, 1.20) |
| New Jersey | 1.08 (1.01, 1.15) | 1.13 (1.05, 1.20) | 1.00 (0.94, 1.07) | REF | 0.97 (0.88, 1.07) | 0.95 (0.79, 1.14) |
| Oregon | 0.98 (0.88, 1.09) | 0.94 (0.86, 1.03) | 0.99 (0.90, 1.08) | REF | 0.72 (0.27, 1.88) | N/A |
| **Pooled** | 1.01 (0.98, 1.05) | 1.00 (0.97, 1.02) | 0.99 (0.97, 1.01) | REF | 1.00 (0.98, 1.02) | 1.02 (1.00, 1.05) |
| All models adjusted for maternal age, LMP month, and LMP year; cases matched 1:4 to controls based on maternal race, education, and county | | | | | | |

| Supplemental Table 8. Odds ratios and 95% confidence intervals for categorical percentile model | | | | | | | |
| --- | --- | --- | --- | --- | --- | --- | --- |
|  | <2.5% | 2.5-10% | 10-25% | 25-75% | 75-90% | 90-97.5% | >97.5% |
| California | 1.00 (0.94, 1.07) | 0.99 (0.95, 1.03) | 1.01 (0.98, 1.04) | REF | 1.02 (0.99, 1.05) | 1.01 (0.97, 1.05) | 1.00 (0.94, 1.06) |
| Florida | 0.97 (0.90, 1.05) | 0.98 (0.93, 1.03) | 0.98 (0.95, 1.02) | REF | 1.01 (0.98, 1.05) | 0.99 (0.95, 1.04) | 1.08 (1.00, 1.17) |
| Georgia | 0.97 (0.88, 1.06) | 1.02 (0.96, 1.08) | 0.98 (0.94, 1.02) | REF | 0.98 (0.94, 1.02) | 1.04 (0.98, 1.10) | 1.00 (0.92, 1.09) |
| Kansas | 1.08 (0.88, 1.33) | 1.07 (0.94, 1.23) | 1.03 (0.92, 1.14) | REF | 0.94 (0.85, 1.04) | 1.06 (0.94, 1.20) | 1.01 (0.82, 1.25) |
| New Jersey | 1.01 (0.89, 1.14) | 0.99 (0.92, 1.07) | 1.03 (0.97, 1.10) | REF | 0.95 (0.90, 1.01) | 0.95 (0.88, 1.04) | 0.90 (0.79, 1.02) |
| Oregon | 0.98 (0.79, 1.21) | 0.95 (0.83, 1.08) | 1.04 (0.95, 1.15) | REF | 1.04 (0.94, 1.14) | 1.04 (0.92, 1.18) | 1.22 (1.01, 1.48) |
| **Pooled** | 0.99 (0.95, 1.03) | 0.99 (0.97, 1.02) | 1.00 (0.98, 1.02) | REF | 1.00 (0.98, 1.02) | 1.01 (0.99, 1.04) | 1.02 (0.98, 1.06) |
| All models adjusted for maternal age, LMP month, and LMP year; cases matched 1:4 to controls based on maternal race, education, and county | | | | | | | |
